# Scarcity of ecosystem services: an experimental manipulation of declining pollination rates and its economic consequences for agriculture

**DOI:** 10.7717/peerj.2099

**Published:** 2016-07-05

**Authors:** Harpinder Sandhu, Benjamin Waterhouse, Stephane Boyer, Steve Wratten

**Affiliations:** 1 School of the Environment, Flinders University, Adelaide, Australia; 2 Bio-Protection Research Centre, Lincoln University, Lincoln, New Zealand; 3 Environmental and Animal Science, Unitec Institute of Technology, Auckland, New Zealand

**Keywords:** Brassica, Economic value, ES, Pollination scarcity, Seed production, Pollination rate

## Abstract

Ecosystem services (ES) such as pollination are vital for the continuous supply of food to a growing human population, but the decline in populations of insect pollinators worldwide poses a threat to food and nutritional security. Using a pollinator (honeybee) exclusion approach, we evaluated the impact of pollinator scarcity on production in four brassica fields, two producing hybrid seeds and two producing open-pollinated ones. There was a clear reduction in seed yield as pollination rates declined. Open-pollinated crops produced significantly higher yields than did the hybrid ones at all pollination rates. The hybrid crops required at least 0.50 of background pollination rates to achieve maximum yield, whereas in open-pollinated crops, 0.25 pollination rates were necessary for maximum yield. The total estimated economic value of pollination services provided by honeybees to the agricultural industry in New Zealand is NZD $1.96 billion annually. This study indicates that loss of pollination services can result in significant declines in production and have serious implications for the market economy in New Zealand. Depending on the extent of honeybee population decline, and assuming that results in declining pollination services, the estimated economic loss to New Zealand agriculture could be in the range of NZD $295–728 million annually.

## Introduction

An increasing world population and current food distribution and consumption patterns will require a 60% increase in global food production by 2050 (United Nations Environment Programme, 2012; FAO, 2013). These global trends are challenging agriculture to increase its contribution to the meeting of the food and nutritional security requirements of a growing population. Achieving those goals is possible when agricultural practices recognise and enhance ecosystem services (ES) from farmland and minimise trade-offs between production and the environment (United Nations Environment Programme, 2011; United Nations Environment Programme, 2014). Such ES include the benefits obtained from agriculture, such as food, fibre, pollination, nutrient cycling, etc. (Sandhu, Crossman & Smith, 2012a; Sandhu, Nidumolu & Sandhu, 2012b, Sandhu et al., 2015; Sandhu & Wratten, 2013; Wratten et al., 2013). There is therefore a need to acknowledge the contribution of ecosystem functions that have potential not only to improve ES in general, but also to sustainably enhance food production in agroecosystems. Despite significant advances in the scientific understanding of the consequences of degradation of different types of ES in agriculture, current policies at national and global level continue largely to ignore the value of ES contributions to the achievement of food and nutritional security (FAO, 2013). Thus, it is critical to understand marginal changes in ES and their economic consequences, in order to identify appropriate policy responses to avert further losses in ES and to reinstate their contribution to agriculture (Sandhu, Nidumolu & Sandhu, 2012b). Here, we consider the example of pollination services provided by honeybees (*Apis mellifera*) as a key ES in agriculture and estimate economic consequences of their decline through experimental manipulation of pollination rates in a New Zealand brassica crop. It is highly unlikely that all pollinators will disappear at once so incremental change in the status and value of ES (in this case, pollination services) is a better measure to develop appropriate responses at the policy level.

Pollination services are one of the key ES associated with agro-ecosystems, as one-third of crop species worldwide require animal pollinators (Prescott-Allen & Prescott-Allen, 1990; Roubik, 1995; Klein et al., 2007; Potts et al., 2010; Winfree, Bartomeus & Cariveau, 2011; Albrechdt et al., 2012; Breeze et al., 2014). There are two main methods to assess the economic value of pollination. First, by assessing the total value of crops that are dependent on pollinators. Second, by assessing plants’ pollinator dependency rates (% fruit or seed set by insect pollinators) and calculating the value of the crop that is attributed to particular rates. Both these methods have been used in a number of studies to estimate the value of pollination at local, national and global scales (Matheson, 1987; Robinson, Nowogrodzki & Morse, 1989; Southwick & Southwick, 1992; Costanza et al., 1997; Costanza et al., 2014; Pimentel et al., 1997; Losey & Vaughan, 2006; Allsopp, De Lange & Veldtman, 2008; Gallai et al., 2009; Brittain & Potts, 2011; Lautenbach et al., 2012; Garibaldi et al., 2013). However, a more direct estimation of the economic value of pollination based on the experimental manipulation of pollination rates is rare. This approach can have two major advantages. Firstly, it provides a better estimate of the economic value of pollination services as opposed to attributing total value of the crop to pollinators. Secondly, changes in the pollination rates can provide estimates of changes in ecosystem function and the corresponding economic value of ES for appropriate policy responses (Perrings et al., 2011). For example, a policy response to changes in the population of pollinators can encourage formulating policy actions through increasing investments in research, thereby benefitting other associated ES (FAO, 2006; FAO, 2009; Wratten et al., 2012).

ES are declining worldwide due to the expansion and intensification of agro-ecosystems (Matson et al., 1997; Tilman, 1999; Millennium Ecosystem Assessment, 2005; TEEB, 2010; Intergovernmental Science-Policy Platform on Biodiversity and Ecosystem Services, 2010). For example, the expansion of monoculture, associated habitat destruction and extensive use of pesticides (insecticides, fungicides and herbicides) in agriculture is creating a global decline of pollinators (Watanabe, 1994; Nabhan & Buchmann, 1997; Biesmeijer et al., 2006; Winfree et al., 2009) and may be reducing pollinator efficiency (Potts et al., 2010; Garibaldi et al., 2009; Rader et al., 2009). Because food security relies so heavily on animal pollinators, pollination services have a substantial role in reducing world hunger (Kleijn et al., 2011; FAO, 2013). To meet the growing demand for food and fibre, humans have become increasingly reliant on managed honeybee colonies. However, the spread of the parasitic mite (*Varroa destructor* Anderson and Trueman) and associated diseases (Mondet et al., 2014), prophylactic pesticide use, agricultural intensification and lower market prices for honey and other apiary products are resulting in declines in the populations of both wild and managed honeybees globally (Delaplane & Mayer, 2000; Steffan-Dewenter, Potts & Packer, 2005). In New Zealand, the economic impact of the varroa mite alone is estimated to be in the range of US $300–600 million to agriculture annually over the next 35 years (Simpson, 2002). Hive pesticides are available but resistance to these can be a problem (Mozes-Koch et al., 2000), and non-managed colonies located in forests and other ‘natural’ areas are unlikely to be treated in this way, for logistical reasons.

In New Zealand, some arable land produces high-value seed crops, for which farmers rent European honeybee hives every year to provide pollination (Ministry of Agriculture and Forestry, New Zealand, 2006), adding to the cost of production. Any major reduction in populations of pollinators will lead to severe losses to the seed industry through production declines and the costs of renting more hives (Matheson, 1987). Therefore, we simulated the consequences of reduced pollination rates of this insect using a pollinator exclusion approach in pak choi fields to examine: (i) the consequences of reduced pollination rate on seed yield in the field and (ii) the resulting economic impacts of this experimentally manipulated scarcity of pollination services. There are about 507,000 registered colonies of honeybees in New Zealand. About half this number is used to provide pollination services to farmers/growers for the crops that require pollination by honeybees (Ministry of Agriculture and Forestry, New Zealand, 2006; Ministry for Primary Industries, New Zealand, 2014). We assume that the varying rates of pollination used in this study (devised by varying the period of the exposure of the inflorescence to pollinators) mimic reductions in the number of managed honeybees.

## Materials and Methods

### Pollination system

The study was conducted in four commercial fields producing seeds of *Brassica campestris* L., chinensis group (pak choi), in the Canterbury Plains, New Zealand. The study was carried out on private land, with permission from the owners. Two fields were producing open-pollinated seeds and two were producing hybrid seed. The genus *Brassica* (Cruciferae: Brassicaceae) includes many crop species that are commercially important for oil seeds, leaf and stem vegetables, condiments, forage, fodder and green manure (Friend, 1985). For most brassicas, although flowers are hermaphrodite, there is some degree of self-incompatibility and pollination by insects such as bees (Apoidea) or flies is usually required. Although open-pollinated (self-fertlising) crops set seed through a combination of selfing and by pollinators, hybrid (pollinator-dependent) crops, which have separate male and female plants, require pollinators to transfer pollen between them. There are three bee and four fly species, which are the key pollinators of this crop in the study region (Rader et al., 2009). Although, all these species are efficient in transferring pollen, managed honeybees are more effective due to their high population, which leads to higher rates of visitation (Rader et al., 2009).

### Experimental plots

In each of the four fields, the crop was sown with a precision drill with an inter-row spacing of 30 cm and with 5 cm between plants in the rows. The two fields with open-pollinated crops were sown on 10 October 2005 and the two hybrid fields were sown on 25 September 2005 for female rows and on 2 October 2005 for male plants, with a female to male ratio of 3:2. The mean size of the fields was c.10 ha. In each, an experimental plot (10 × 10 m) was set up 20 m from the nearest field edge. In each plot there were five treatments arranged in a randomized block design. These comprised plants whose inflorescences were covered with 1 mm white mesh bags for one, two, three or four weeks, or uncovered during the four-week flowering period. Each treatment was replicated six times with a 2 m distance between replicates. For the analysis, the treatments were considered in terms of the proportion of time the plants were exposed to pollinators (pollination rates; 0–no pollinators i.e., bagged for the entire pollination period of 4 weeks; 0.25–bagged for three weeks; 0.50–bagged for 2 weeks; 0.75–bagged for 1 week; and 1.00: no bagging).

All hybrid crops began flowering during the first week of December 2005 and were available for pollination for four weeks. During this period, New Zealand seed producers (arable crops) bring in honeybee hives at a density of 4 hives ha^−1^ and at a cost of NZD $120/hive/1 month. All the experimental plots in each field were at least 50 m from hives. At the end of flowering, experimental plants were individually removed from the plots, labeled with replicate and field information and placed in paper bags. They were dried in the shade at room temperature for four weeks and the following information was recorded:

Seeds per pod: Seeds were counted in 50 randomly selected pods for each replicate.
Seed yield per plant: Seeds for each plant were separately threshed and cleaned using a sieve (2 mm). The seeds were stored in paper bags, which were then weighed individually to calculate seed yield (g dry weight) per plant.
Proportion of unfertilised pods per plant: For each treatment, the numbers of fertilised and unfertilised pods were recorded.

Data were analysed by randomised-block ANOVA followed by LSD pair-wise comparisons of the two pak choi types (open-pollinated and hybrid) using GENSTAT 7.2. A log_10_ transformation was used to normalize the variance for seed weight.

### Economic impacts

Most flowering plants in New Zealand except grasses require pollinators for pollination and honeybees are the most effective pollinators of commercial crops (Rader et al., 2009). Data for New Zealand on types of crops that require pollinators, their area, total production in tons and market value in NZD (2013) was obtained from the Ministry of Primary Industries, New Zealand website. Data on area and production were not available for vegetable, clover and rye grass seed, however, their market values were available. Therefore, for these three crop types, those values were used in our calculations. Plants’ pollinator-dependency rates and proportion of honeybees as pollinators for each crop were obtained from the literature (Klein et al., 2007; Gallai et al., 2009). We selected a list of 18 crops including fruits, vegetable and pasture seed where honeybees account for 90% of the pollinators. The market value of each crop was then used to calculate the value of pollination services. The economic value attributed to pollination by honeybees was obtained from the following Eq. (1).

(1) $$TEV_{psc}=V_{mc}\times D_{ic}\times P_{hbc}$$

Where,

*TEV_psc_* = Total economic value attributed to pollination services by bees in each crop
*V_mc_* = Market value of the crop
*D_ic_* = Insect dependency ratio of the crop
*P_hbc_* = Proportion of insect pollinators that are honeybees in each crop

Seed yield data in the current study were used to estimate the economic impact of a reduction in the current pollination services in New Zealand due to decreases in pollination rates (a surrogate for a reduction in pollinator numbers) for both hybrid and open-pollinated crops. Percentage decrease in yield was calculated from the seed yield data at each of the four pollination rates.

Changes in the economic value of pollination services provided by honeybees was calculated according to the marginal reduction in yield and economic value due to varying pollination rates by using Eq. 2.

(2) $$\Delta TEV_{psc}=TEV_{psc}-(TEV_{psc}\times V_{prc})$$

Where,

Δ*TEV_psc_* = Change in the value of pollination services by bees in each crop
*V_prc_* = Percentage change in the economic value at different pollination rates in each crop

We calculated change in the economic value for each crop at different pollination rates. The aggregated economic value of pollination services provided by honeybees at a national scale was calculated by aggregation of the total value assigned to each crop. Change in total economic value for the main pollinator-reliant crops in New Zealand was also calculated at different pollination rates.

## Results

We present our findings across two main themes that illustrate the economic consequences of reduced pollination rates in New Zealand agriculture. First, we identified any change in seed yield, seeds per pod and proportion of unfertilised pod in the experimental plots as a consequence of changing pollination rates. Then, the economic impact of the varying pollination rates was extrapolated to the main 18 pollination-dependent crops in New Zealand.

### Experimental assessment

#### Seed yield per plant

**Figure 1:**
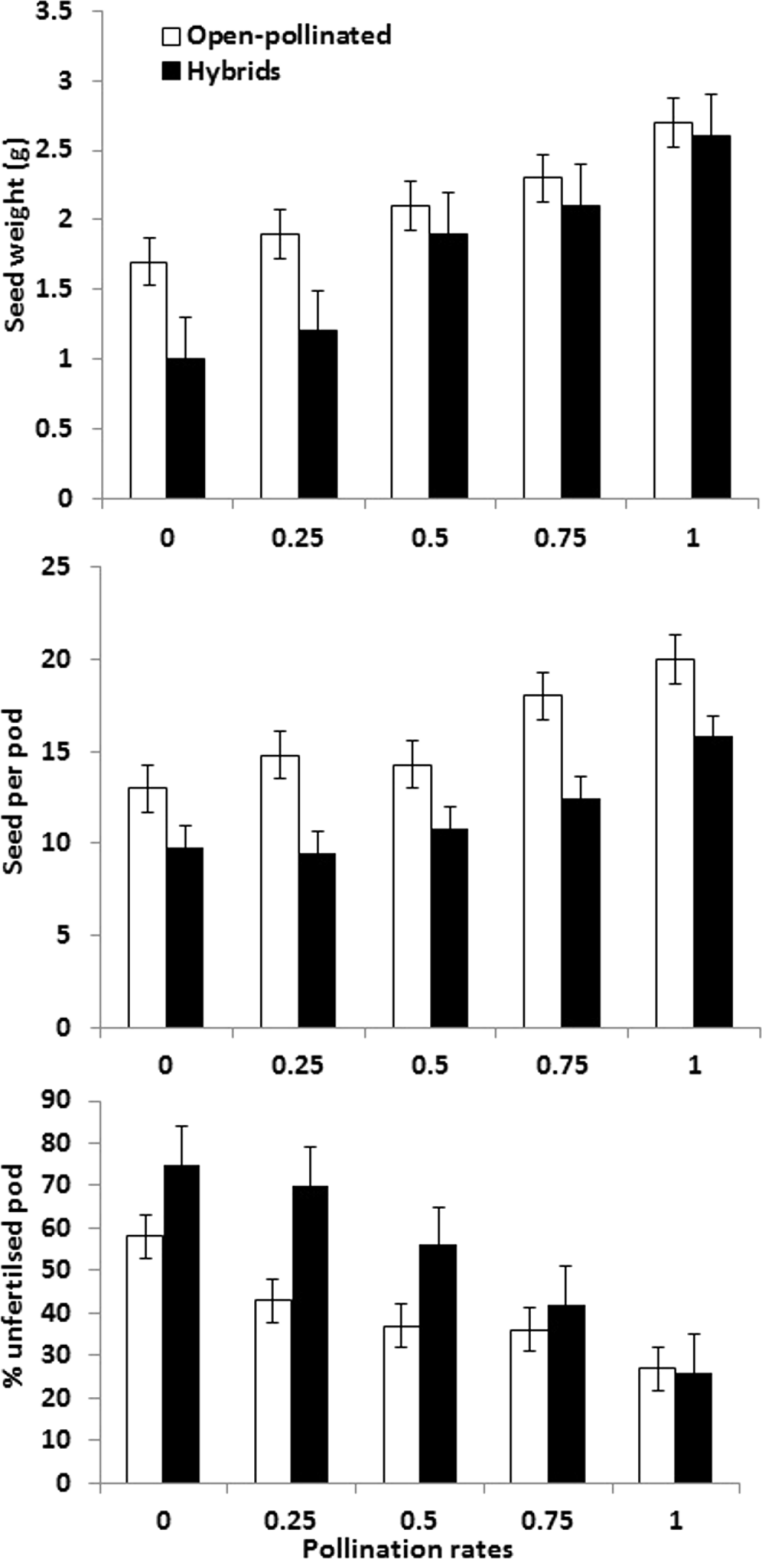
Seed yield per plant, seeds per pod and percentage of unfertilised pods per plant in hybrid and open-pollinated crops (mean plus SE).

There was a significant difference between the open-pollinated and the hybrid crops for seed yield with no manipulation (*p* = 0.002, Table 1). At lower pollination rates (0 and 0.25), the former produced significantly higher yield than did the hybrids. There was a greater increase in seed yield per plant in the hybrid crop with increasing proportion of time that pollinators had access (Fig. 1) with the maximum increase at 0.50 pollinator exclusion. In open-pollinated crops there was an increase with each 0.25 increase in pollinator access but the rate of increase in seed yield (Fig. 1) was not as high as that found in the hybrid crop. For the latter, there was a reduction of 60% in seed yield compared to 37% in the open-pollinated ones in the complete absence of pollinators (0–Pollination rate). At 0.25 pollination rate, hybrid crops suffered a 52% decline in seed yield whereas open-pollinated showed a 26% decline. At 0.50, hybrids lost 24% and open-pollinated lost 22% of their potential seed yield. At 0.75 pollination rate, hybrids and open-pollinated fared equally, with reductions in yield of 15 and 16%, respectively.

**Table 1:** Summary of the results produced by ANOVA for seed weight (g), seeds per pod and percentage of unfertilised pods in two hybrids and two open-pollinated fields.

	Seed weight (g)	Seeds per pod	% Unfertilised pods
Hybrid	1.7 ± 0.3	11.7 ± 1.1	53.6 ± 9.0
Open-pollinated	2.2 ± 0.2	16.08 ± 1.3	40 ± 5.1
Significance	*p* < 0.001	*p* < 0.001	*p* < 0.001

#### Seeds per pod

Open-pollinated crops produced significantly more seeds per pod than did the hybrid ones (*p* = 0.003). Seed number per pod increased significantly with increasing pollination rates for both hybrid and open-pollinated crops (*p* < 0.001) (Fig. 1).

#### Proportion of unfertilised pods per plant

The proportion of unfertilized pods per plant was significantly higher in the hybrid crop than in pollinated ones at lower pollination rates (0, 0.25 and 0.50; *p* = 0.025). There was a declining proportion of unfertilised pods in both hybrid and open-pollinated crops with increasing pollination rates (Fig. 1).

### Estimated economic losses from reduced pollination rates—an extrapolation

**Figure 2:**
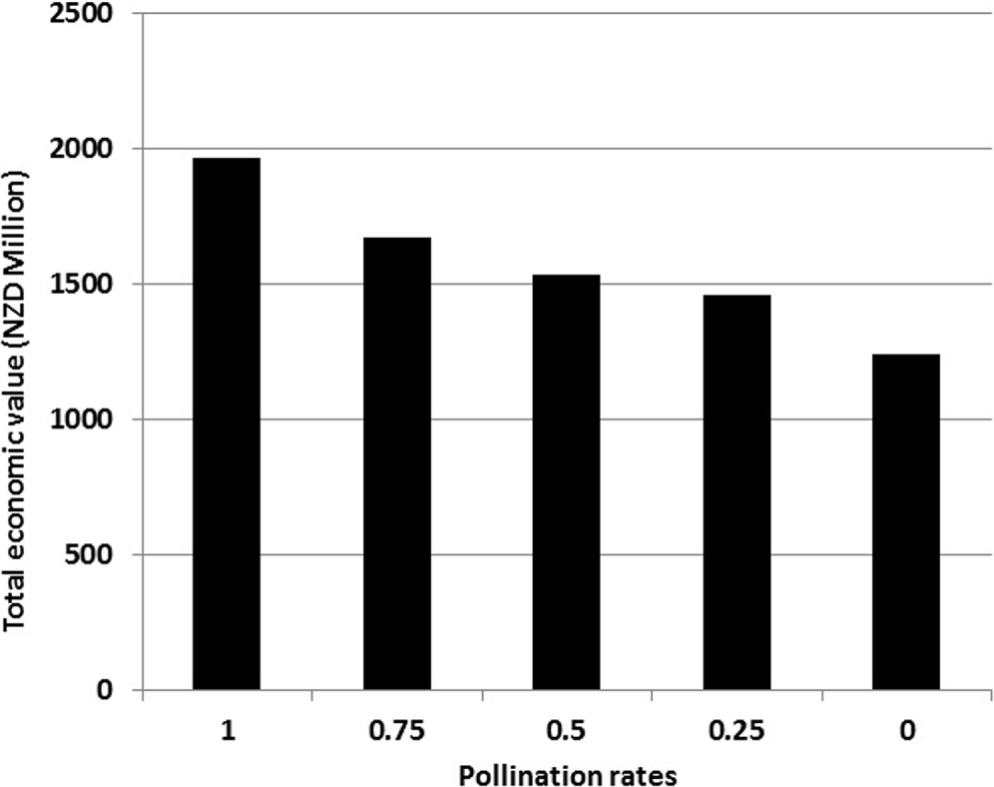
Total economic value attributed to pollination services by honeybees in New Zealand agriculture at different pollination rates.

The total market value of the 18 crop types, to which the experimental results were extrapolated, examined in this study is NZD $2.55 billion annually (Table 2). Based on insect pollinator dependency ratios for each crop and the proportion of pollinators that are honeybees in each case (Klein et al., 2007; Gallai et al., 2009), we estimated the economic value of honeybee pollination services for each crop (Table 2) at varying pollination rates. We used only the data obtained from declines in seed yield from open-pollinated crops in this study, as the 18 crops used in Table 2 are open-pollinated. The total economic value of pollination services provided by honeybees for these 18 crops was NZD $1.96 billion annually. Figure 2 provides the annual total economic value at different pollination rates for these New Zealand crops. The economic loss to New Zealand agriculture could be in the range of NZD $295–728 million/year depending on the extent of future changes in pollination provided by honeybees.

**Table 2:** Area, production and market value of pollinator dependent crops in New Zealand. Insect dependency, proportion of honeybee pollination and economic value of crops at different pollination rates is also given.

Crops	Area (Ha)	Total volume (Tons)	Total value (M NZD)	Insect dependency	Proportion of pollinators that are honeybees (90%)	Value dependent on honeybees pollination (100%) (M NZD)	Value at pollination rates (0%) (M NZD)	Value at pollination rates (25%) (M NZD)	Value at pollination rates (50%) (M NZD)	Value at pollination rates (75%) (M NZD)
Kiwifruit	12,263	380,520	992	0.95	0.85	848.16	534.34	627.63	661.56	720.93
Apples	8,372	606,261	1,010	1	0.9	909	572.67	672.66	709.02	772.65
Green bean	1,500	20,000	63	0.25	0.22	14.17	8.93	10.48	11.05	12.04
Tomato	877	90,000	127	0.05	0.04	5.71	3.60	4.22	4.45	4.85
Blueberries	700	2,450	33	0.65	0.58	19.30	12.16	14.28	15.05	16.40
Cherries	646	2,535	32	0.65	0.58	18.72	11.79	13.85	14.60	15.91
Pears	441	4,381	9	0.7	0.63	5.67	3.57	4.19	4.42	4.81
Orange	406	11,762	17	0.05	0.04	0.76	0.48	0.56	0.59	0.65
Apricots	332	3,283	17	0.7	0.63	10.71	6.74	7.92	8.35	9.10
Peaches	328	2,903	11	0.6	0.54	5.94	3.74	4.39	4.63	5.04
Nectarines	307	3,644	14	0.6	0.54	7.56	4.76	5.59	5.89	6.42
Plums	217	2,413	7	0.7	0.63	4.41	2.77	3.26	3.43	3.74
Boysenberries	204	3,100	5	0.65	0.58	2.92	1.84	2.16	2.28	2.48
Strawberries	170	6,500	26	0.2	0.02	0.52	0.32	0.38	0.40	0.44
Raspberries	150	945	3	0.65	0.58	1.75	1.10	1.29	1.36	1.49
Vegetable seed			82	0.65	0.58	47.97	30.22	35.49	37.41	40.77
Clover seed			41	0.65	0.58	23.98	15.11	17.74	18.70	20.38
Rye grass seed			70	0.65	0.58	40.95	25.79	30.30	31.94	34.80
**Total**	26,913	1,140,697	**2,559**			**1,968.23**	**1,239.98**	**1,456.49**	**1,535.22**	**1,673**

## Discussion

There are two major outcomes of this study that provide insights into the relationships between marginal change in ES and their economic impacts. First, the impact of changing pollination rates on crop yield and differences between crop types were identified. Second, the economic impacts of varying pollination rates in New Zealand agriculture were estimated.

### Marginal change in pollination services

Past pollination studies have used cages or bagging to completely exclude pollinators and study the effect on seed yield, seed per pod etc. (Langridge & Goodman, 1975; Williams, 1986; Free, 1993). However, the consequences of varying pollinator rates on yield of these types of experiments have rarely been analysed. It is clear that pollinator populations are in a state of decline in managed landscapes (Kremen, Williams & Thorp, 2002; Potts et al., 2010; Breeze et al., 2014). Also, varroa mite can seriously reduce managed and wild honeybee colonies, imposing serious economic losses (Simpson, 2002). Our results indicate that if pollinators are very rare or absent from a cropped area, open-pollinated crops could provide a better alternative as their decline in yield in the current work was up to 37% compared to hybrids where, the decline could be as high as 60% (Fig. 2). However, hybrid crops are almost always of higher value than open pollinated varieties of the same crop in market. There would thus be a financial loss if a hybrid crop is simply replaced. Such relative ‘improvements’ still do not fully address the serious implications of pollinator losses for New Zealand and world agriculture. Globally, there has been an increasing trend in the cultivation of pollinator-dependent crops since 1961 (Aizen & Harder, 2009; Gilbert, 2016; Intergovernmental Science-Policy Platform on Biodiversity and Ecosystem Services, 2016). These crops have also produced yield increases by using honeybees (Garibaldi et al., 2009). However, in some parts of the world, especially USA and Europe, recent declines in managed honeybee colonies are serious (Potts et al., 2010; Intergovernmental Science-Policy Platform on Biodiversity and Ecosystem Services, 2016). The manipulated reduction in exposure to pollinators in the current study serves as an indicator of the potential consequences of future honeybee population declines.

Our logic in this study assumes that a 50% decrease in exposure to pollinators in terms of flowering time is equivalent to a 50% decrease in the availability of managed bee hives in farmland. We argue that if farmers use a fixed number of bee hives to maximise production of crops that depend on pollinators, then any reduction in hive number will impact on yield, as observed in this study (Fig. 1). However, although we manipulated access by pollinators to the experimental crops, it remains unknown whether the ambient pollinators populations could give maximum possible pollination rates in the controls. It follows that if more pollinators had been present, control yields may have been higher and the proportional reductions in pollination rates achieved here would have probably been even higher. Also, the availability and pollinator-dependency of adjacent crop and non-crop plants in the vicinity of the experiment is unknown and that could have reduced the number of pollinators visiting the un-covered, control plots. It is also important to note that the insect dependency rates, i.e., how many pollinators are honey bees, were obtained from global datasets, which also includes several New Zealand based studies (Klein et al., 2007). However, further investigation is required to confirm change in seed yield under varying pollination rates and varying population of pollinators (e.g., by different number of beehives) per field in an experimental setup.

### Economic impacts of loss of pollinators

**Figure 3:**
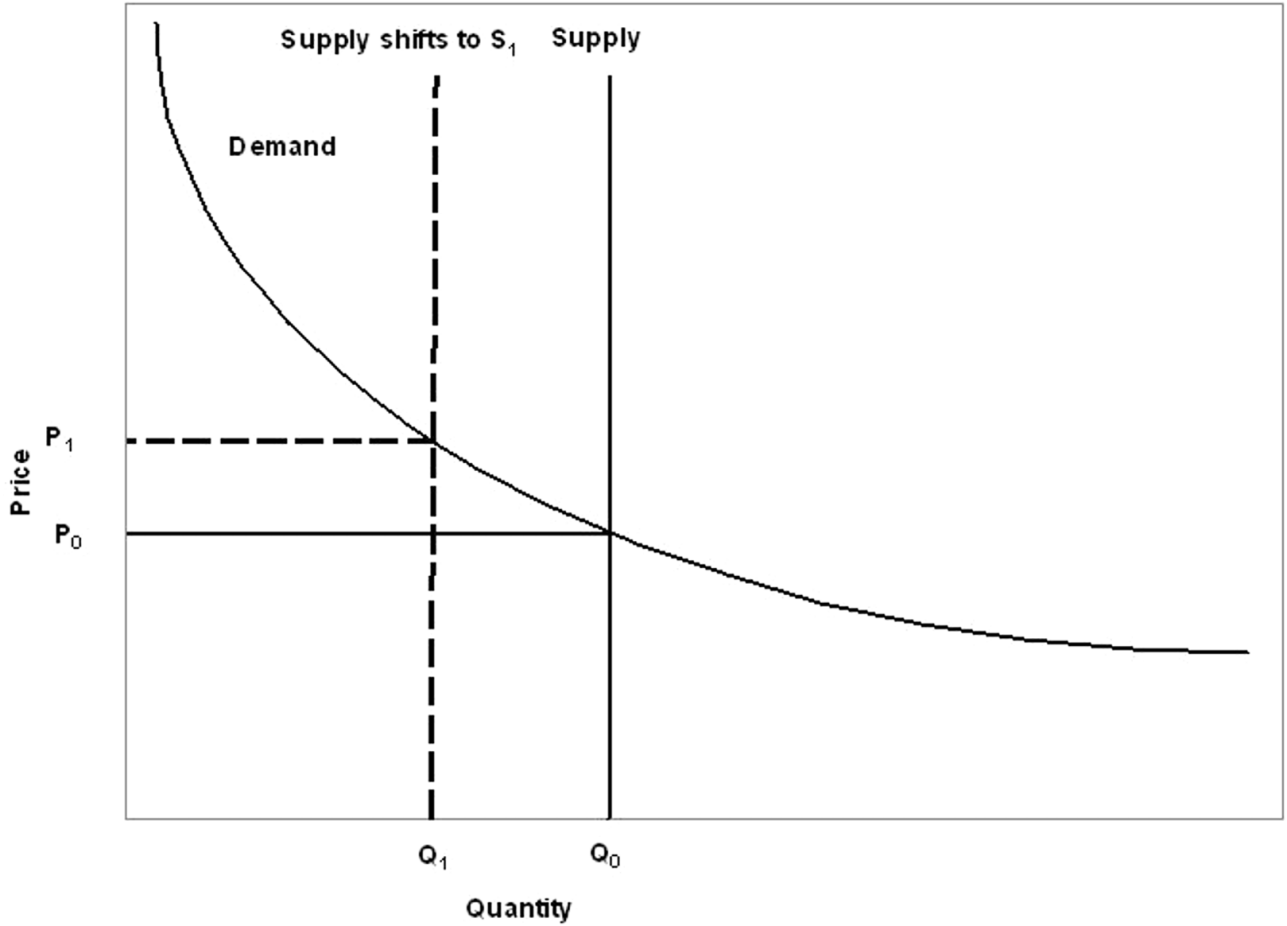
Supply and demand curves for pollination services. Scarcity of pollination rates shifts the supply curve to left to the new supply curve S_1_. This will shift price from P_0_ to P_1_ quantity Q_0_ to Q_1_ [adapted from Costanza et al. (1997) and Kevan & Phillips (2001)].

Pollinator declines have been reported worldwide (Williams, 1984; Falk, 1991; Buchmann & Nabhan, 1998; Kevan & Phillips, 2001; Kremen, Williams & Thorp, 2002; Millennium Ecosystem Assessment, 2005; Winfree et al., 2009; Potts et al., 2010). These have rarely resulted in complete failure of crops, but more frequently resulted in reduced yield. The scarcity of pollination services on farmland shifts the supply and demand function in the market (Southwick & Southwick, 1992). As most ES are not substitutable or are substitutable only to an extent, the demand and supply curve for pollination services will resemble Fig. 3, with increased prices to consumers at existing levels of demand, while producers sell fewer products but at higher prices. The supply curves of ES are usually vertical (Costanza et al., 1997; Costanza et al., 2014) as their supply is not affected by the forces of the economy. They are, however, influenced by those economically-driven changes which reduce the biodiversity that provides ES. In the case of pollination services, managed honeybee hives can be increased or decreased in number on farmland to change the extent of this service, so if the supply curve shifts from S_0_ to S_1_, the price will increase from P_0_ to P_1_ (Fig. 3). This can have further economic implications for beekeepers and food markets (Barrionuevo, 2007; Teagasc, 2007). Globally, there is a need to grow more food to meet the demand of growing populations and changes in consumption patterns. To help meet these challenges, there is a need to reduce reliance on honeybee colonies to provide pollination. Ecosystems with a high rate of delivery of ES with minimal ecosystem dis-services (Zhang et al., 2007; Wratten et al., 2013) in agricultural landscapes can assure continuous supply of pollination services by wild pollinator species (Kremen et al., 2004; Winfree et al., 2007). For example, favouring wild pollinator species, this can provide continuous supply of pollination services (Kremen et al., 2004; Winfree et al., 2007) thereby providing high rate of delivery of ES with minimal ecosystem dis-services (Wratten et al., 2013).

Modifying existing agricultural systems to enhance ES requires a range of mechanisms such as payments for ES (PES; Wunder, 2005). There are already agri-environment schemes that target enhancement of pollination services due to the recognition of their high economic value (Ricketts et al., 2004; Albrechdt et al., 2007). Adoptions of these schemes depend upon policy changes, as they require financial investment at national and farm level. Set-aside land or reducing the intensity of farming may result in a higher provision of pollination services but only if such practices are informed by sound ecological science (Kleijn et al., 2011). However, a consequence may be that farmers may suffer foregone benefits for example lose some of their crop yields. PES schemes should consider these options, including paying for the foregone income that otherwise would have been generated by farmers (FAO, 2007).

## Conclusion

The experimental approach in this work has been used to investigate the impact of scarcity of ES (pollination services) by hierarchical exclusion of pollinators from flowers of pak choi and explore the possible yield and economic consequences of future declines in populations of managed honeybees. This study provides an insight into the relationship between marginal change in ES and its economic consequences. Declines in managed and wild pollinator populations have been attributed to multiple stressors including the varroa mite (Steffan-Dewenter, Potts & Packer, 2005), habitat change through agricultural intensification (Breeze et al., 2014) and the associated use of agrochemicals (Van der Sluijs et al., 2013). A 25% or greater loss in pollination rates is a likely scenario in areas where pollinator numbers have been steadily decreasing. This work shows that such decrease could have a major economic impact on the agricultural industry in New Zealand. Although the expected losses can be mitigated to some extent through the deployment of commercial beehives, the cost of renting these is also increasing with increased production and maintenance difficulties (Ministry of Agriculture and Forestry, New Zealand, 2006). There is a need to further investigate the drivers of pollinator decline to aid appropriate policy responses. Maintaining current pollination rates in the future with the sole commercial beehives is a major challenge for agriculture. Decrease in pollinator populations will inevitably lead to significant changes in the breeding of crop varieties, cropping patterns and practices, the consequences of which are largely unknown.

## Additional Information and Declarations

### Data Deposition

The following information was supplied regarding data availability: All raw data is included in the manuscript.
